# MRI Atlas of the Human Deep Brain

**DOI:** 10.3389/fneur.2019.00851

**Published:** 2019-08-27

**Authors:** Jean-Jacques Lemaire, Antonio De Salles, Guillaume Coll, Youssef El Ouadih, Rémi Chaix, Jérôme Coste, Franck Durif, Nikos Makris, Ron Kikinis

**Affiliations:** ^1^Service de Neurochirurgie, CHU Clermont-Ferrand, Université Clermont Auvergne, Centre National de la Recherche Scientifique, Engineering School SIGMA Clermont, Clermont-Ferrand, France; ^2^Department of Neurosurgery, Radiation Oncology, HCOR Neuroscience, São Paulo, Brazil; ^3^Service de Neurologie, Centre National de la Recherche Scientifique, CHU Clermont-Ferrand, Université Clermont Auvergne, Engineering School SIGMA Clermont, Clermont-Ferrand, France; ^4^Surgical Planning Laboratory, Center for Morphometric Analysis, A. Martinos Center for Biomedical Imaging, Harvard Medical School, Massachusetts General Hospital, Brigham and Women's Hospital, Boston, MA, United States; ^5^Robert Greenes Distinguished Director of Biomedical Informatics, Brigham and Women's Hospital, Boston, MA, United States; ^6^Computer Science Department, Fraunhofer MEVIS, University of Bremen, Bremen, Germany

**Keywords:** atlas, MRI, human, thalamus, hypothalamus, subthalamus, deep brain, stereotaxis

## Abstract

Mastering detailed anatomy of the human deep brain in clinical neurosciences is challenging. Although numerous pioneering works have gathered a large dataset of structural and topographic information, it is still difficult to transfer this knowledge into practice, even with advanced magnetic resonance imaging techniques. Thus, classical histological atlases continue to be used to identify structures for stereotactic targeting in functional neurosurgery. Physicians mainly use these atlases as a template co-registered with the patient's brain. However, it is possible to directly identify stereotactic targets on MRI scans, enabling personalized targeting. In order to help clinicians directly identify deep brain structures relevant to present and future medical applications, we built a volumetric MRI atlas of the deep brain (MDBA) on a large scale (infra millimetric). Twelve hypothalamic, 39 subthalamic, 36 telencephalic, and 32 thalamic structures were identified, contoured, and labeled. Nineteen coronal, 18 axial, and 15 sagittal MRI plates were created. Although primarily designed for direct labeling, the anatomic space was also subdivided in twelfths of AC-PC distance, leading to proportional scaling in the coronal, axial, and sagittal planes. This extensive work is now available to clinicians and neuroscientists, offering another representation of the human deep brain ([https://hal.archives-ouvertes.fr/] [hal-02116633]). The atlas may also be used by computer scientists who are interested in deciphering the topography of this complex region.

## Introduction

The term “deep brain” (DB) describes the combination of subcortical structures including the mesencephalon. It is anatomically a highly complex region with clinical importance in a number of diseases. Several specialized atlases have been created to orient deep brain interventions. The atlases of the human deep brain most used in clinical neurosurgery relies on histological studies (1–4). They are both, anatomic books, and stereotactic atlases. They are co-registerable with patient brains through landmarks; such as the widely used anterior (AC) and posterior (PC) white commissures. Other proportional grid systems derived from landmarks, are also still largely used (5, 6). These atlases provide probabilistic coordinates of structural related functional targets. Rigid registration of an atlas to a patient's brain MRI refines the quality of probabilistic targeting (7). This approach is called indirect stereotactic identification of structures (2), because the structures of interest were not directly visible before the existence of the MRI.

The deep brain geometry limited variability coupled with the relative facility to construct proportional diagrams made the indirect visualization approach used worldwide in stereotactic and functional neurosurgery, including radiosurgery (8). Yet an increasing number of surgical teams transitioned to use both, MRI landmarks and/or directly visualization of targeted structures for implantation of DB stimulation (DBS) (9–15). We proposed direct targeting of pallidal structures visualized on MRI without the use of AC-PC referencing (16), and since used this approach routinely (17).

Recent advances of MRI technologies have resulted in a dramatic increase in both, spatial resolution and contrast resolution, consequently the ability to identify clinically relevant structures in the deep brain. Our group begun to manually segmenting the DB using *ex vivo* high field MRI in the early 2000's (18). Because of its complex architecture, it took over 10 years to comprehensively label the majority of relevant structures. We reported intermediate stages of this atlas over the years (19–22). Herein we present a clinical MRI Deep Brain Atlas (MDBA) built from a unique anatomic specimen offering for the first time the most advanced version with detailed volumetric representation. Though mainly developed to identify structures of the deep brain on MRI for neurosurgical practices, it also offers to neuroscientists another representation of the topographic organization of the deep brain.

## Materials and Methods

### Specimen, Raw Image Data, and Initial Contouring of Structures

The brain specimen was obtained from a 65 year-old male subject who died of non-neurological cause. It was studied following our institutional rules and guidelines. After long term fixation in 10% formaldehyde, a block measuring about 60-mm in each direction was scanned at 4.7 Tesla (Bruker, Ettlingen, Germany) with a 3D T1-weighted spin echo sequence (about 14 h of acquisition), resulting in 250 μm isotropic voxels (256^3^ matrix). The image data was initially manually contoured and labeled using a neurosurgical software (Iplan, BrainLab, Munich, Germany). MRI cartography and labeling relied on the analysis of different signals and patterns of the deep brain structures (19). The signal intensity of a voxel reflects the microarchitecture, i.e., cell density, and anisotropy of bundles of axons (the higher number of anisotropic bundles, the lower signal), as well as the cells contents, notably the ferromagnetic load of neurons (the higher ferromagnetic charge, the lower signal). In addition, at the resolution available in our data set, the common separation of brain tissue into white and gray matters is not binary in the deep brain. For instance, at large scale (centimetric) the thalamus is made of gray matter; at small scale (millimetric) the thalamus is made of gray matter nuclei such as the ventromedial posterior nucleus, of white fascicles such as the mammillothalamic fascicle, and of mixed structures such as intralaminar nuclei or the reticular nucleus crossed by numerous white matter fibers. The cartography was performed structure by structure, starting from the most readily identifiable ones, such as the subthalamic nucleus. In parallel to the progressive mapping of the 4.7-Tesla data set, the updated version was tested in clinical practice for direct targeting in functional neurosurgery as an neuroanatomic aid (23, 24). The different nuclei of the hypothalamus were parcellated into different structures according to proportional topography and structural connectivity (21, 25).

### Building of the MRI Deep Brain Atlas

**Figure 1:**
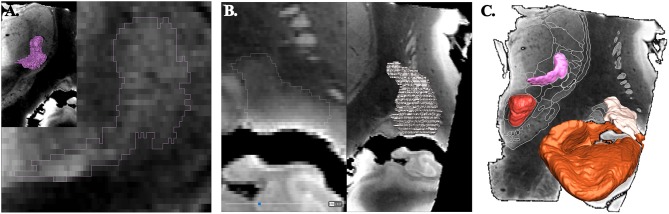
Principle of contouring and voxel objects (frontal view; coronal slices). **(A)** ventrocaudal medial nucleus of thalamus (pink). **(B)** retrolenticular-reticularoïd zone (light beige). **(C)** smoothed surfaces of voxel objects, ventrocaudal medial nucleus of thalamus, retrolenticular-reticularoïd zone, red nucleus (red), and hippocampal formation (light brown).

The objects, i.e., the anatomic structures, were exported as surface (stl format) from the surgical software. Surfaces were transformed in binary maps for voxel-based representation and co-registered with a modified MRI data set (Thermo Scientific™ Amira™, v 6.4, Hillsboro, OR, USA). The raw image data set were realigned along AC-PC line, slices being resampled accordingly; leading to a new image data set of 0.125 × 0.125 × 0.256 mm^3^ voxels (512 × 512 × 256 matrix; 8-bit grayscale ranging from 0 to 255). The resampled MRI images were cleaned (removed of cerebrospinal-fluid spaces, vessels and nerves of the subarachnoid space; Photoshop CC, Adobe, San Jose, CA, USA,) and then filtered (unsharp masking and sigmoid intensity remapping; 16-bit grayscale; Thermo Scientific™ Amira™, v 6.4, Hillsboro, OR, USA). All imported objects were manually re-segmented accounting to the new high geometric resolution and highest contrast between adjacent structures using multi-objects contouring tools (Thermo Scientific™ Amira™, v 6.4, Hillsboro, OR, USA) (Figure 1A). Contours of unnamed structures were identified and labeled during this process (Figure 1B). A unique color (HSV color model) was attributed to each object. The surface representation of voxel objects was smoothed with a Gaussian filter (Thermo Scientific™ Amira™, v 6.4, Hillsboro, OR, USA) (Figure 1C).

### Labeling of Structures

**Figure 2:**
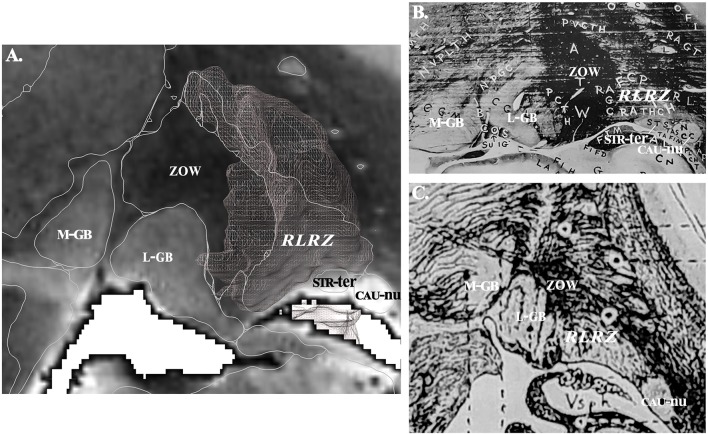
**(A)** Contouring and labeling of the retrolenticular reticularoid zone (*RLRZ*; the voxel object is overlaid) on MRI slice (see MDBA plate 10); medial geniculate body, M-GB; lateral geniculate body, L-GB; stria terminalis, STR-ter; caudate nucleus, CAU-nu. **(B)** Same region according to Riley (36); the *RLRZ* is visible (histologic preparation) but not identified. **(C)** Same region according to Dejerine (26); the RLRZ is visible (artist drawing) but not identified.

The structures were labeled according to clinically known classical names (1, 2, 26–37) and ontologies (38–40). These structures were essentially nuclear, i.e., where neuronal bodies are concentrated; in addition, small white matter fascicles embedded were included (e.g., thalamic fascicle), but we did not label the large capsule fascicles, namely the internal, external and extreme capsules. Complementary information, such as homonyms and French names were also added. Acronyms were created to reduce the text size of labels on plates. Structures not precisely identified or still unnamed were detailed and labeled according to the location and the aspect on MRI. For instance: (i) the retrolenticular reticularoid zone was observed laterally to the area or zone of Wernicke, hence in a retrolenticular position, Because of its reticular appearance (low signal intensity) it was named reticularoid (Figure 2); (ii) the subthalamic tegmental field covered the historical Forel's H field, and was segregated into anterior, dorsal, medial, lateral and central zones; (iii) the area of reticular appearance, i.e., with an apparent low density of cells, which is placed posteriorly and below the pallidum, was named the posterior subpallidal area. The information is available as Supplementary Table.

The structures were also specified according to four subregions of the deep brain, although these subdivisions are still not formally set (33, 40), namely the hypothalamus, the thalamus, the subthalamus or prethalamus and the telencephalon (Supplementary Table). The labeling was not fully extensive, as we focused on structures identifiable on MRI for the thalamus, subthalamus, and telencephalon, or inferred from diagrams for the hypothalamus (e.g., the suprachiasmatic and supraoptic nuclei were not separated).

### MDBA Plates

For each MRI slice and related maps, structural (MRI slice) and topographic (maps) data were distributed on a double page or plate.

**Figure 3:**
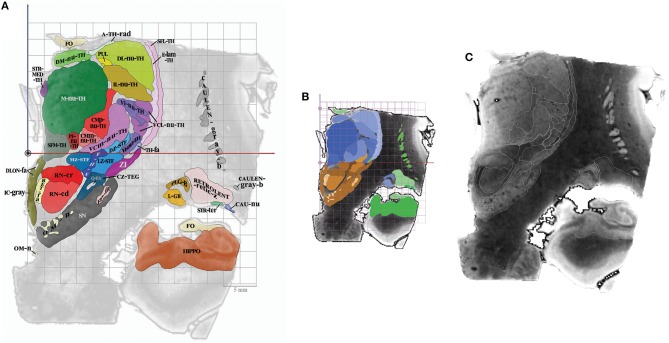
**(A)** Colored surfaces and acronyms, plate CR8 (millimeter scale), the ACPC line is depicted as a black circle. **(B)** Same plate, colors are specified according to subregions (proportional and millimeter scales); thalamus (blue gradation), subthalamus (brown gradation) and telencephalon (green gradation). **(C)** Same plate, MRI slice and white contours of structures (no scale).

Topographic maps were created from cross sections of objects intersecting with cutting planes (Thermo Scientific™ Amira™, v 6.4, Hillsboro, OR, USA), displaying colored surface and contours of structures overlaid on MRI slices (Figure 3). The MRI slices were not scaled, as they were assumed to be used for direct comparison with patient MRI datasets. Furthermore, the aspect of signal of structures is very similar to that observed in images routinely used in clinical practice, even if the latter have a lower spatial resolution, which is supramillimetric. The contours of structures were overlaid (white line) on each MRI slice, facilitating the identification of structures on the patients' individual imagery. The contrast of each MRI slice was enhanced by automatic adjustment of tones (Photoshop CC, Adobe, San Jose, CA, USA), in a slice by slice fashion, minimizing heterogeneity of signals due to the presence of extremely high (white) and low (black) values within the volume of acquisition (a legacy of the original MRI acquisition).

**Figure 4:**
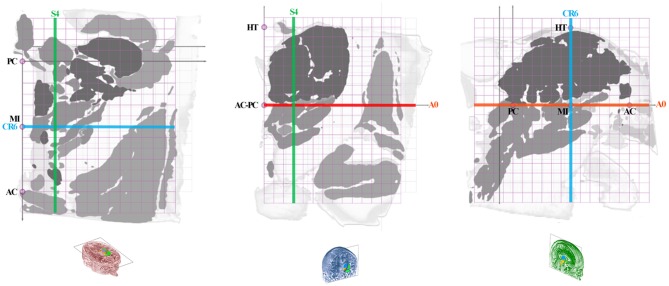
Position of slices (colored lines: axial, red; coronal, blue; sagittal, green) on the axial ACPC plan (A0-ACPC; left row), coronal MI plan (CR6-MI; intermediate row) and 9-mm lateral sagittal plan (S4; right row); proportional grid (purple) in 12th of ACPC distance; HT, thalamus height.

The anatomic space, scaled in millimeters and oriented along vertical and horizontal AC-PC plans, was subdivided in twelfth of AC-PC distance according to Guiot et al. (5), Benabid et al. (6), leading to proportional scaling in the coronal (C), axial (A), and sagittal (S) planes (Figure 4). AC-PC distance was rounded to 27 mm; 1/12th of AC-PC was rounded to 2.25 mm; the midpoint between AC and PC (MI) was rounded to 13.5 mm. The height of the thalamus was 18 mm. The proportional grid system numbers were used to name the slices and related maps. Hence, for one unique location in a plane, both absolute (overlay of absolute millimeter distance grid) and relative (overlay of proportional distance grid) positions were available. Three particular sections served as reference positions. The axial section going through the AC-PC horizontal plane was named A0-AC-PC; sections above that plane were named superior (AS), and below, inferior (AI). The coronal section going through AC (perpendicular to AC-PC horizontal plane) was named C0-AC. All plates in front of AC were named CF, whereas the ones located posteriorly (or rear) to AC were named CR; at the MI point, the coronal section was named CR6-MI (the 6th coronal plane posterior to AC); at PC, CR12-PC (the 12th coronal plane posterior to AC). The sagittal section going through the vertical AC-PC plane was named S0-ACPC.

On each plate whatever the orientation, one MRI slice and related position graphs and maps, were arranged for localization and comparison purposes. The relative position of any given MRI slice and related maps was overlaid on proportional grids on A0-ACPC, CR6-MI, or S4, according to its orientation. Two related maps of the MRI slice were displayed: the first is the map of structures at the specific location with the contours and labels (acronyms), on which is overlaid a millimetric grid (absolute location); the second map is made of the same structures but colored according to the four subregions, including the overlay of a proportional grid as well. This second map was colored using luminance gradients of the specific color of the subregion as follows. The hypothalamus was colored in yellow, the thalamus in blue, the subthalamus in brown, and the telencephalon in green.

## Results

Twelve hypothalamic, 39 subthalamic, 36 telencephalic and 32 thalamic structures were identified, contoured and labeled (*n* = 119; Supplementary Table). Nineteen coronal, 18 axial, and 15 sagittal MRI plates were created (*n* = 52; Table 1). The acronyms were classified in alphabetic order by subregion. The 52 plates generated for this study can be found in the https://hal.archives-ouvertes.fr/ [hal-02116633].

**Table 1:** MDBA plates.

**Coronal**			**Axial**			**Sagittal**		
**Acronym**	**Relative position (1/12th of ACPC)**	**Location (mm)**	**Acronym**	**Relative position (1/12th of ACPC)**	**location (mm)**	**Acronym**	**Relative position (1/12th of ACPC)**	**Location (mm)**
CF2	2 in front of AC	−4.5	AS8	8 above ACPC	18	S0-ACPC	0 of ACPC	0
CF1	1 in front of AC	−2.2	AS7	7 above ACPC	15.5	S1	1 of ACPC	2.3
CO-AC	0 of AC	0	AS6	6 above ACPC	13.5	S2	2 of ACPC	4.5
CR1	1 rear to AC	2.2	AS5	5 above ACPC	11.3	S3	3 of ACPC	6.8
CR2	2 rear to AC	4.5	AS4	4 above ACPC	9	S4	4 of ACPC	9
CR3	3 rear to AC	6.7	AS3	3 above ACPC	6.8	S5	5 of ACPC	11.3
CR4	4 rear to AC	9	AS2	2 above ACPC	4.5	S6	6 of ACPC	13.5
CR5	5 rear to AC	11.2	AS1	1 above ACPC	2.3	S7	7 of ACPC	15.8
CR6-MI	6 rear to AC	13.5	A0-ACPC	0 of ACPC	0	S8	8 of ACPC	18
CR7	7 rear to AC	15.7	AI1	1 below ACPC	−2.3	S9	9 of ACPC	20.3
CR8	8 rear to AC	18	AI2	2 below ACPC	−4.5	S10	10 of ACPC	22.5
CR9	9 rear to AC	20.2	AI3	3 below ACPC	−6.8	S11	11 of ACPC	24.8
CR10	10 rear to AC	22.5	AI4	4 below ACPC	−9	S12	12 of ACPC	27
CR11	11 rear to AC	24.7	AI5	5 below ACPC	−11.3	S13	13 of ACPC	29.3
CR12-PC	12 rear to AC	27	AI6	6 below ACPC	−13.5	S14	14 of ACPC	31.5
CR13	13 rear to AC	29.2	AI7	7 below ACPC	−15.8			
CR14	14 rear to AC	31.4	AI8	8 below ACPC	−18			
CR15	15 rear to AC	33.7	AI9	9 below ACPC	−20.3			
CR16	16 rear to AC	35.9						

## Discussion

The MDBA with 119 structures and 52 plates provides an extensive 3D MRI structural analysis of the human deep brain mainly for clinical applications, but also researchers interested in direct visual identification of neuroanatomical structures. The simple principle of cartography from reconstructed slices of one anatomic specimen without destruction of tissue greatly facilitates the 3D structural analysis, which is also dramatically improved by high spatial resolution with infra millimetric voxels. Although the result of parcellation according to T1-weighted contrast harvested a lot of data, further approaches using others MRI contrast, such as inversion-recovery sequences, or multimodal imaging with DTI, should refine the information.

The large scale maps of MDBA with 250-μm side voxels is compatible with the recent human DTI data sets with isotropic voxels of 400 μm (41) up to 60 μm (42), as well as with high resolution probabilistic atlases (43, 44) and could help in the labeling process of the deep brain. Indeed the MDBA gives high level of structural details of white and gray matter structures substantially enhancing the current structural knowledge within this region. Although it can be used both at the individual level and in series, it is intrinsically a detailed data set of a unique specimen which must interpreted in this strict context as a topological descriptor of the deep brain architecture. Anyway this topological descriptor, could be the support of advanced probabilistic atlases enabling to integrate the variability, still not mastered, of the deep brain, through large cohorts of subjects.

Our approach has shown that it is feasible to identify the details of individual MRI anatomy. Whereas, the atlas-with proportional scales is still largely used for stereotactic targeting, nevertheless there must be kept in mind such unsolved issues as inhomogeneity of ontologies, weak cross-correspondences between atlases (45) and between set of slices within atlases questions (20). On the other hand it can be assumed that machine learning approaches (46) could significantly enhance these anatomical uncertainties, therefore dramatically change paradigms to solve these challenges. This is expected as the learning databases are rapidly becoming stronger. For instance the MDBA could be used in the work flow of learning methods including decision-based approaches whether supervised or not (47–49) to interpret the results. In the interim, the MDBA can assist significantly those who are willing to better master the deep brain architecture, which is particularly important for clinicians implanting devices in the deep brain. In this latter condition, practitioners can use the atlas like classical histological atlases from the proportional grid plates, and at the same time they can adjust or specify directly the targets from the MRI plates. Furthermore, the MDBA is of considerable value to study injured and deformed brains as indirect methods are unreliable due to the hampered landmarks in the injured brain (50). The MDBA also improves scientific knowledge of the deep brain structural aspect as revealed by current MRI. In this sense, it fuels the panoply of MRI-based brain atlases used for research and clinical purposes, notably in computer science (see e.g., https://en.wikibooks.org/wiki/SPM/Atlases). Furthermore, the MDBA creates a link to pioneering data (see Supplementary Table), which otherwise would remain into oblivion. Moreover, the MDBA serves as a new tool in the continuous effort of mastering the structural and functional anatomy of the human brain using either direct or indirect methods of cartography. Last but not least, it can also be used for teaching, learning and training purposes, taking advantage of current publicly available free website-based programs (https://www.openanatomy.org/).

## Data Availability

One dataset generated for this study can be found in the [https://hal.archives-ouvertes.fr/] [hal-02116633].

## Author Contributions

J-JL designed and realized the MDBA and wrote the first draft of the manuscript. AD contributed substantially to the interpretation of anatomical data of the MDBA and wrote sections of the manuscript. GC, YE, RC, JC, and FD contributed substantially to the interpretation of anatomical data of the MDBA. NM and RK contributed to the conception the work and wrote sections of the manuscript. All authors contributed to manuscript revision, read and approved the submitted version.

### Conflict of Interest Statement

The authors declare that the research was conducted in the absence of any commercial or financial relationships that could be construed as a potential conflict of interest.
