# Supplementary material for: MRI Atlas of the Human Deep Brain
Source: Front Neurol. 2019 Aug 27;10:851. doi: 10.3389/fneur.2019.00851 (PMC6718608; doi:10.3389/fneur.2019.00851)
Supplement: Supplementary file 1 [file Data_Sheet_1.pdf]

Supplementary Material

Supplementary Table

References of French (FR) books: Laget, P. Elements de Neuro-Anatomie Fonctionnelle. Masson. Paris, 1973 / Talairach, Jean, M David, P Tournoux, H Corredor, and T Kvasina. Atlas d'anatomie Stéréotaxique. Repérage Radiologique Indirect Des Noyaux Gris Centraux Des Régions Mésencéphalo-Sous-Optiques et Hypothalamiques de l'homme. Paris: Masson et Cie, 1957 / Dejerine, J. Anatomie Des Centres Nerveux (Tomes 1 and 2). Rueff et Cie. Paris, 1901 / Duvernoy, HM, E-A Cabanis, M-T Iba-Zizen, J Tamraz, and J Guyot. Le Cerveau Humain: Surfaces, Coupes Sériées Tridimentionelles et IRM. Springer-Verlag. Paris Berlin Heidelberg New York Londres Tokyo Hong Kong Barcelone Budapest, 1992 / Guillain, Georges, and Ivan Bertrand. Anatomie Topographique Du Système Nerveux Central. Paris: Masson et Cie, 1926.

**MRI Deep Brain Atlas**		**NeuroNames (ID)**	**Foundational Model of Anatomy (ID)**	**Terminologia Anatomica 1998 (ID)**	**Terminologia neuroanatomica 2017**	**usages, classical and French terminologies**		
**Acronym**	**MDBA name**	http://braininfo.rprc.washington.edu	https://www.unifr.ch/ifaa/Public/EntryPage/TA98%20Tree/Alpha/All%20KWIC%20EN.htm		http://fipat.library.dal.ca/wp-content/uploads/2017/02/FIPAT-TNA-Front-Matter.pdf	others usages	FR (in Laget; Talairach et al, Déjerine, Duvernoy, Guillain & Bertrand)	In Riley, Olszewski & Baxter, Schaltenbrand & Bailey and Nieuwenhuys, Voogd & Van Huijzen
**hypothalamus**								
**ot**	optic tract	optic tract (460)	optic tract (62046)	tractus opticus (A14.1.08.404)	tractus opticus		tractus optique; bandelette optique (rétrochiasmatique)	fasciculus opticus
**dm-nu-hypot**	dorsomedial nucleus of hypothalamus	dorsomedial nucleus of the hypothalamus (397)	dorsomedial nucleus of hypothalamus (62331)	nucleus dorsomedialis (A14.1.08.922)	nucleus dorsomedialis hypothalami		noyau dorso-médian de l'hypothalamus	nucleus dorso-medialis hypothalami; nucleus hypothalamicus dorso-medialis; nucleus centralis infundibuli

**inf-nu-hypot**	infundibular nucleus of hypothalamus	arcuate nucleus of the hypothalamus (395)	arcuate nucleus of hypothalamus (63329)	nucleus arcuatus (A14.1.08.923)	nucleus arcuatus; semilunaris; infundibular	arcuate, infundibular, periventricular nucleus	noyau arqué (infundibulaire) de l'hypothalamus	infundibular nucleus
**l-hypot-a**	lateral hypothalamic area	lateral hypothalamic area (426)	lateral hypothalamic area (62030)	area hypothalamica lateralis (A14.1.08.929)	areae hypothalamicae lateralis	lateral area	aire hypothalamique latérale; zone latérale de l'hypothalamus	nucleus hypothalamicus lateralis or area hypothalamica lateralis
**i-hypot-a**	lateral intermediate hypothalamic area	n.a.				overlapping of the lateral hypothalamic area and the tuberomammillary nucleus of hypothalamus	n.a.	
**mb**	mammillary body	mammillary body (412)	mammillary body (74817)	corpus mammillare (A14.1.08.402); area hypothalamica posterior (A14.1.08.933)	corpus mammillare	nucleus lateral, medial, intercalatus; ; area mammillary	corps mamillaire; tubercule mamillaire	corpus mammillare; albicans; candescans; ganglion mammilare
**post-nu-hypot**	posterior nucleus of hypothalamus PH	posterior hypothalamic area (420)	dorsal nucleus of hypothalamus (77685)	nucleus dorsalis hypothalami (A14.1.08.921)	nucleus posterior hypothalami	dorsal nucleus	aire hypothalamique postérieure & aire hypothalamique dorsale	nucleus hypothalamicus posterior or area hypothalamica posterior
**pv-nu-hypot**	paraventricular nucleus of hypothalamus	paraventricular nucleus of the hypothalamus (427)	periventricular nucleus (no ID number)	nucleus periventricularis (A14.1.08.924)	nucleus paraventricularis hypothalami	filiformis	noyau paraventriculaire, filiforme, de l'hypothalamus	nucleus paraventricular
**po-nu-hypot**	preoptic nucleus of hypothalamus	preoptic area (377)	preoptic area (62213)	area preoptica (A14.1.08.407); area hypothalamica rostralis (A14.1.08.902)	area hypothalamica anterior (chiasmatica)	preoptic (medial, lateral, anterior) nucleus of hypothalamus; prothalamus	noyau préoptique de l'hypothalamus	area preoptica
**so-nu-hypot**	suprachiasmatic nucleus & supra-optic nucleus, of hypothalamus	suprachiasmatic nucleus (384); supraoptic nucleus (385)	suprachiasmatic nucleus (67883); supra-optic nucleus (62317)	nucleus suprachiasmaticus (A14.1.08.911); nucleus supraopticus (A14.1.08.912); area hypothalamica rostralis (A14.1.08.902)	nucleus suprachiasmaticus & nucleus supraopticus	suprachiasmatic nucleus & supra-optic (dorsomedial, dorsolateral, ventromedial) nucleus, of hypothalamus	noyau suprachiasmatique & noyau supra-optique, de l'hypothalamus	nucleus ovoideus or suprachiasmaticus; nucleus supraopticus (hypophyseus, tangentialis) hypothalami

**tm-nu-hypot**	tuberomammillary nucleus of hypothalamus	tuberomammillary nucleus (427)	tuberomammillary nucleus (62335)	nucleus tuberomammillaris (A14.1.08.932)	nucleus tuberomammillaris	tuberomamillaris; mamilloinfundibularis	noyau tubéro-mammillaire	nucleus tubero-mammilaris
**vm-nu-hypot**	ventromedial nucleus of hypothalamus	ventromedial nucleus of the hypothalamus (398)	ventromedial nucleus of hypothalamus (62332)	nucleus ventromedialis hypothalami (A14.1.08.928)	nucleus ventromedialis hypothalami	ventromedial nucleus; principal tuberis or infundibular medialis	noyau ventro-médian de l'hypothalamus	nucleus hypothalamicus ventro-medialis or ventralis tuberis cinerei; nucleus infundibularis medialis
**subthalamus**								
**az-stf**	anterior zone of subthalamic tegmental field	n.a.				anteromedial to the H field	n.a.	
**au**	area U	n.a.				ventrolateral and inferior region of the tegmental pontomesencephalic reticular formation; and more specifically of the pedunculopontine nucleus	n.a.	U-Field (Riley, Ziehen)
**br-conj**	brachium conjunctivum	superior cerebellar peduncle, superior cerebellar peduncle of the pons (1736)	superior cerebellar peduncle (72495)	pedunculus cerebellaris superior (A14.1.05.006)	brachium conjunctivum	superior cerebellar peduncle (& commissure of Wernekink, horshoe commissure)	pédoncule cérébelleux supérieur; brachium conjunctivum (& commissure de Wernekink, entrecroisement du pédoncule cérébelleux supérieur)	tractus cerebello-rubralis; tractus cerebello-tegmentalis cerebralis
**br-i-coll**	brachium of inferior colliculus	brachium of the inferior colliculus (480)	brachium of inferior colliculus (71114)	brachium colliculi inferioris (A14.1.06.012)	brachium colliculi inferioris	auditory pathway (med-sial geniculate body)	bras conjonctival inférieur ou postérieur	brachium (pedunculus) colliculi inferioris; caudalis; posterius; inferius
**br-s-coll**	brachium of superior colliculus	brachium of the superior colliculus (474)	brachium of superior colliculus (72417)	brachium colliculi superioris (A14.1.06.013)	brachium colliculi superioris	visual pathway (lateral geniculate body)	bras conjonctival supérieur ou antérieur	brachium (pedunculus) colliculi superioris; rostralis; anterius; superius

**c-teg-tr**	central tegmental tract	central tegmental tract (of the midbrain, pons) (2204)	central tegmental tract (83850)	tractus tegmentalis centralis (A14.1.05.325)	tractus tegmentalis centralis		faisceau central de la calotte (thalamo-olivaire)	tractus tegmentalis centralis
**cz-teg**	central zone of tegmentum	n.a.				ventral to the H field; it contains the fasciculus (bundle) Q of Sano	n.a.	
**dlon-fa**	dorsal longitudinal fascicle	dorsal longitudinal fasciculus (1573)	dorsal longitudinal fasciculus of medulla (72617)	fasciculus longitudinalis posterior, dorsalis (A14.1.04.114)	fasciculus longitudinalis posterior	dorsal or posterior longitudinal fasciculus; dorsal longitudinal fasciculus of Schultze (Schuetz or Schüts)	faisceau longitudinal dorsal; faisceau de Schütz, Schüts	fasciculus longitudinalis periependymalis or periacquaeductalis; tegmentalis dosrsalis; tractus bulbo-thalamicus; periventricular fibre system
**dz-stf**:	dorsal zone of subthalamic tegmental field	n.a.				dorsal to the H field	n.a.	
**f-h-f**	Forel's H field	field H (440)	nucleus of field h (62037)	nucleus campi medialis [H] (A14.1.08.704)	campi medialis	containing the prelemniscal radiations; medial field	champs H, prérubrique; radiations de la calotte	area tegmentalis subthlamica; prerubral field; H; medial field; area tegmentalis H
**i-coll**	inferior colliculus	inferior colliculus (476)	inferior colliculus (62404)	colliculus inferior (A14.1.06.014)	colliculus inferior	posterior colliculus	colliculus, tubercule quadrijumeau, inférieur, postérieur	colliculus inferior or caudalis or posterius; corpus quadrigeminium inferius or posterius
**ic-gray**	interbrain central gray	central gray (1584)	n.a.			central gray	substance grise sous-ependymaire	substantia grisea centralis
**inf-pbga**	inferior parabigeminal area	parabigeminal area (1241) and cuneiform nucleus (502)	parabigeminal nucleus (72415)	nucleus parabigeminalis (A14.1.06.320)	nucleus parabigeminalis	the parabigeminal area (Riley) is contained within the nucleus cuneiformis (Olszewski & Baxter)	aire parabigéminale	area parabigemina posterior; area bigemina; area cuneiformis; nuclei cuneiformis and subcuneiformis

**ipn-ni**	interpeduncular paranigrae nuclei	interpeduncular nucleus (522) & paranigral nucleus (1252)	interpeduncular nucleus (72439) & paranigral nucleus (77497)	nucleus interpeduncularis (A14.1.06.313); nucleus paranigralis (A14.1.06.333)	nucleus interpeduncularis; nucleus paranigralis		noyau interpédonculaire & noyau paranigrique; ganglion interpédonculaire	nucleus (corpus, ganglion) intercruralis or interpeduncularis; possibly nucleus medialis tegmento-peduncularis or tegmenti, and area reticulata substantiae nigrae
**l-lem**	lateral lemniscus	lateral lemniscus (609)	lateral lemniscus (72502)	lemniscus lateralis (A14.1.05.317)	lemniscus lateralis		ruban de Reil latéral, lemniscus latéral; ruban de Reil, portion postérieure; ruban de Reil inférieur	lemniscus acusticus, lateralis
**lz-stf**	lateral zone of subthalamic tegmental field	n.a.				lateral to the H field	n.a.	
**m-lem**	medial lemniscus	medial lemniscus (1583)	medial lemniscus (83675)	lemniscus medialis (A14.1.04.111)	lemniscus medialis		ruban de Reil médian; lemniscus médian; ruban de Reil, portion antérieure); lemniscus	lemniscus sensibilis, medialis
**mlon-fa**	medial longitudinal fascicle	medial longitudinal fasciculus (1588)	medial longitudinal fasciculus of medulla (72618)	fasciculus longitudinalis medialis (A14.1.04.113)	fasciculus longitudinalis medialis	medial longitudinal fasciculus	bandelette longitudinale postérieure; faisceau longitudinal postérieur	tractus longitudinalis medialis; fasciculus commissurae posterioris; fasciculus longitudinalis dorsalis or posterioris
**mz-stf**	medial zone of subthalamic tegmental field	n.a.				medial to the H field	n.a.	
**om-n**	oculomotor nerve (III)	oculomotor nerve (488)	n.a.	nervus oculomotorius [III] (A14.2.01.007)	nervus oculomotorius	common oculomotor nerve	nerf moteur oculaire (commun)	nervus oculomotorius communis; third cerebral(cranial) nerve

**pag**	periaqueductal gray	periaqueductal gray (510)	central gray-grey substance (83134)	substantia grisea centralis (A14.1.06.321)	substantia grisea centralis	central grey substance of midbrain	subtance grise périaqueducale (& périventriculaire)	substantia centralis grisea; anulus aquaeductus; stratum griseum centrale, mesencephali
**per-ped-nu**	peri peduncular nucleus	peripeduncular nucleus (520)	peduncular nucleus of pons (77123)	nucleus peduncularis (A14.1.05.207)	nucleus peripedoncularis	peduncular nucleus	noyau péri-pédonculaire	nucleus peripeduncularis dorsalis (nppd); processus lateralis substantiae nigrae
**ped-p-fi**	pedunculopontin fibrae	fasciculus obliquus crucis cerebri (1569)	n.a.			part of the fascicle circumligatus	faisceau en écharpe de Féré	fascicle circumligatus; obliquus cruris or crus cerebri
**ped-p-nu**	pedunculopontin nucleus	pedunculopontine nucleus (509)	pedunculopontine tegmental nucleus (72429)	nucleus tegmentalis pedunculopontinus (A14.1.06.336)	nucleus tegmentalis pedunculopontinu s	pedunculopontine tegmental nucleus	n.a.	
**p-g**	pineal gland	pineal gland (297)	pineal body/gland (62033)	glandula pinealis (A11.2.00.001)	glandula pinealis	corpus pineale; epiphysis; nervus pinealis	organe (ou glande) pinéal(e); épihyse	conarium
**q-fa**	fascicle	n.a.				fascicle Q of Sano	n.a.	fasciculus Q (Sano); fibrae mediales sustantiae nigrae
**rn**	red nucleus	red nucleus (and its capsule) (505)	red nucleus (62407)	nucleus ruber, magnocellularis, parvocellularis and posteromedialis (A14.1.06.323)	nucleus ruber		noyau rouge	nucleus ruber tegmenti; nucleus rotondus subthalamo-peduncularis
**rn-cd**	red nucleus, caudal part	n.a.					noyau rouge	pars caudalis
**rn-cr**	red nucleus, cranial part	n.a.					noyau rouge	pars rostralis
**ret-fa**	retroflexus fascicle	fasciculus retroflexus of the interbrain (461)	habenulo-interpeduncular tract (72400)	tractus habenulointerpeduncularis, fasciculus retroflexus (A14.1.08.502)	tractus habenulointer-peduncularis; bundle of Meynert	habenulointerpeduncular tract; fasciculus, fascicle of Meynert	faisceau rétroflexe (rétrofléchi, rétroflexus) de Meynert; faisceau habénulo-pédonculaire	fasciculus habenulo-interpeduncularis; tractus retroflexus; tractus tegmentalis nuclei habenulae

**s-coll**	superior colliculus	superior colliculus (473)	superior colliculus (62403)	colliculus superior (A14.1.06.015)	colliculus superior	anterior colliculus	colliculus, tubercule quadrijumeau, supérieur, antérieur	colliculus superior or caudalis or oralis; corpus quadrigeminium superius or anterius
**sn**	substantia nigra	nucleus substantia nigra (2487)	substantia nigra (67947)	substantia nigra (A14.1.06.111)	substantia nigra	SN	subtance noire; locus niger	locus or nucleus niger; nucleus pigmentosus subthalamo-peduncularis; substance of Von Soemmering
**spi-th-fa**	spinothalamic fascicle	spinothalamic tract (2058)	set of spinothalamic fibers (75206)	fibrae spinothalamicae (A14.1.04.138)	tractus spinothalamicus	made of the anterior and lateral spinothalamic fibers of the medulla	faisceau spino-thalamique	lemniscus spinalis; tractus spino-thalamicus; tractus spino-reticulo-thalamicus
**st-nu**	subthalamic nucleus	subthalamic nucleus (and its capsule) (435)	subthalamic nucleus (62035)	nucleus subthalamicus (A14.1.08.702)	nucleus subthalamicus	body of Luys; corpus Luysi; STN	corps de Luys; noyau sous-thalamique	corpus subthalamicum; body of Forel; nucleus hypothalamicus
**sup-pbga**	superior parabigeminal area	parabigeminal area (1241) and cuneiform nucleus (502)	parabigeminal nucleus (72415)	nucleus parabigeminalis (A14.1.06.320)	nucleus parabigeminalis	the parabigeminal area (Riley) is contained within the nucleus cuneiformis (Olszewski & Baxter)	aire parabigéminale	area parabigemina anterior; area bigemina; area cuneiformis; nuclei cuneiformis and subcuneiformis
**teg-pm-ret-for**	tegmental pontomesencephalic reticular formation	formatio reticularis (1223)	reticular formation (77719)	formatio reticularis (A14.1.00.021)	formatio reticularis	tegmental reticular formation of the pons, midbrain and ventral thalamus	formation (substance) réticulaire (réticulée) ponto-mésencéphalique; substance réticulée de la calotte	formatio, substantia, reticularis
**tr-n**	trochlear nerve	trochlear nerve (466)	n.a.	nervus trochlearis [IV] (A14.2.01.011)	nervus trochlearis	pathetic nerve	nerf pathétique ou trochléaire	nervus trochlearis; fourth cerebral(cranial) nerve

**vta**	region of the ventral tegmental area	ventral tegmental area (521)	n.a.			ventral tegmental area (VTA) of Tsai / may include the ento(do)peduncular nucleus/nucleus of ansa lenticularis and the H2 field; area hypothalamica dorsalis (nuclei dorsomedialis, endopeduncularis, ansae lenticularis)	aire ventrale de la calotte; aire de Tsai (noyau ventral de la calotte) (± noyau interpédonculaire & endopedunculaire)	ventral tegmental area; dorsal hypothalamic region or area; area densa
**zi**	zona incerta	zona incerta (438)	zona incerta (62038)	zona incerta (A14.1.08.707)	zona incerta		zona incerta de Forel	nucleus area tegmenti, campi of Forel, infrasensibilis, of Cajal, of zona incerta
**telencephalon**								
**ac**	anterior commissure	anterior commissure (205)	anterior hypothalamic commissure (62053)	commissura anterior (A14.1.08.421)	commissura anterior	AC	commissure blanche antérieure (CA)	commissura anterior or olfactoria or rostralis
**a-lam-gpi**	accessory lamina of internal globus pallidus	accessory medullary lamina (236)	accessory medullary lamina of globus pallidus (62471)	lamina medullaris accessoria (A14.1.09.513)	lamina medullaris accessoria	within Gpi	lame médullaire accessoire	lamina medullaris accessoria (palidi), incompleta
**acc-nu**	accumbens nucleus	nucleus accumbens (277)	nucleus accumbens (61889)	nucleus accumbens (A14.1.09.440)	nucleus accumbens		noyau accumbens; pont de substance grise unissant la partie inférieure des noyaux caudé et lenticulaire; involves the " colliculus du noyau caudé"	fundus striati; involves the colliculus nucleus caudati; nucleus accumbens septi; nucleus parolfactorius lateralis; bed of the stria terminalis
**alen**	ansa lenticularis	ansa lenticularis (444)	ansa lenticularis (62070)	ansa lenticularis (A14.1.08.663)	ansa lenticularis		anse lenticulaire; anse du noyau lenticulaire	ansa lenticularis; part of the "système en peigne"; pars pallido-thalamic of radiate fibers of striatum; the lateral part of the basal forebrain bundle

**alen-ap-nu**	nucleus of ansa lenticularis, anterior part of Riley	nucleus of the ansa lenticularis (436); entopeduncular nucleus (2284)	nucleus of ansa lenticularis (62036); endopeduncular nucleus (77691)	nucleus ansae lenticularis (A14.1.08.919); nucleus endopeduncularis (A14.1.08.918)	n.a.	endo-entopeduncular nucleus	noyau entopédonculaire, noyau de la capsule interne (de cajal); noyau de l'anse lenticulaire	nucleus ansae lenticularis, entopeduncularis, peduncularis; nucleus of Meynert; nucleus subtantia innominate; substantia reticulata hypothalamica
**alen-nu**	nucleus of ansa lenticularis	nucleus of the ansa lenticularis (436); entopeduncular nucleus (2284)	nucleus of ansa lenticularis (62036); endopeduncular nucleus (77691)	nucleus ansae lenticularis (A14.1.08.919); nucleus endopeduncularis (A14.1.08.918)	n.a.	endo-entopeduncular nucleus	noyau entopédonculaire, noyau de la capsule interne (de cajal); noyau de l'anse lenticulaire	nucleus ansae lenticularis, entopeduncularis, peduncularis; nucleus of Meynert; nucleus subtantia innominate; substantia reticulata hypothalamica
**alen-tipp-fa**	ansa lenticularis, tip-pallidal fascicle of Talairach	n.a.				ansa lenticularis	faisceau pallidal de la pointe or fascicule sous-thalamique (part of the ansa lenticularis emerging at the tip of GPi)	n.a.
**amyg**	amygdala	amygdala (237)	amygdala (61841)	corpus amygdaloideum (A14.1.09.402)	corpus amygdaloideum	amygdaloid body or complex	noyau amygdalien; amygdale télencéphalique; complex amygdalien; epistriatum	nucleus amygdaliformis; amygdala; striatum olfactorium; archistriatum; amygdaleum
**amyg-pe**	amygdala posterior extension	n.a.				could be the posterior extension of the basal nucleus of amygdala, an/or the amygdalohippocampal area	n.a.	
**caulen-gray-b**	caudolenticular gray bridges	pontes striatales, pontes striatales (n.a.)	set of caudolenticular gray bridges (77813)	pontes grisei caudatolenticulares; transcapsular grey bridges (A14.1.09.525)	pontes grisei caudatolenticulares	gray laminae bridging the caudate and lenticular nuclei	n.a.	pontes striatales; pontes grisei lenticulares
**cau-nu**	caudate nucleus	caudate nucleus (226)	caudate nucleus (61833)	nucleus caudatus (A14.1.09.502)	nucleus caudatus	caudate	noyau caudé	nucleus caudatus

**d-clau**	dorsal claustrum	dorsal claustrum (252)	n.a.	claustrum (A14.1.09.421)	claustrum dorsale	dorsal part of the claustrum; insular claustrum	avant-mur, claustrum	claustrum; claustrum insulare
**dia-band**	diagonal band	diagonal band (285)	diagonal band (61973)	stria diagonalis (A14.1.09.422)	area diagonalis; diagonal band area	the diagonal band of Broca is visible at the surface of the ventral brain (diagonal gyrus), located between the optic tract and the antererior perforated substance (olfactory tubercle); it is also described within the sublenticular region and is associated with cells referered as the nucleus of the diagonal band of Broca (Riley)	bandelette diagonale de Broca	fasciculus olfactorius; fasciculus substantiae perforatae anterioris; fasciculus septo-amygdalicus; fasciculus hippocampi
**fo**	fornix	fornix (268)	fornix of forebrain (61965)	fornix (A14.1.08.949)	fornix		trigone; fornix	tractus cortico-mammillaris
**fa-g**	fasciolar gyrus	fasciola cinerea (176)	fasciolar gyrus (61921)	gyrus fasciolaris (A14.1.09.233)	fasciola cinerea		gyrus fasciolaris	fasciola cinereum
**fp-fa**	frontopontin fascicle	frontopontine fibers (1326)	set of frontopontine fibers (75223)	fibrae frontopontinae (A14.1.06.106)	tractus frontopontinus		tractus fronto-pontin	fasciculus or tractus fronto-pontinus
**gpe**	external, lateral, globus pallidus	lateral globus pallidus (232)	lateral globus pallidus (61839)	globus pallidus lateralis (A14.1.09.509)	globus pallidus lateralis	globus pallidus external; GPe	globus pallidus, pars lateralis; pallidum externe; deuxième portion du pallidum	globus pallidus, crus II, pars lateralis, externa
**gpi**	internal, medial, globus pallidus	medial globus pallidus (233)	medial globus pallidus (61840)	globus pallidus medialis (pars lateralis and medialis) (A14.1.09.511)	globus pallidus medialis	globus pallidus internal (inner and outer portions); GPi	globus pallidus, pars medialis; pallidum interne; première portion du pallidum; segments superficiel (latéral) et profond (médial)	globus pallidus, crus I, pars mediali, interna

**hippo**	hippocampal formation	hippocampal formation (177)	n.a.	hippocampus (part) (A14.1.09.321)	(most of) the pars retrocommisuralis hippocampi	the precise configuration of the hippocampus is complex; here it is made of the gyrus dentatus, the Ammon's horn, the subiculum complex, the uncus and the fasciolar gyrus; this set represents most strutures of the inferior segment of the intralimbic gyrus	formation hippocampale: gyrus dentatus (circonvolution godronnée; corps godronné)), corne d'Ammon,subiculum, uncus et fasciola cinerea	hippocampus
**inno-sub**	innominate substance	basal (magnocellular) nucleus (275)	substantia innominata (61885)	substantia innominata (A14.1.09.426)	substantia innominata	innominate substance of Reichert; nucleus of Meynert	substance innominée de Reichert	subtantia (regio) innominata (sublenticularis); nucleus planun septale; nucleus basalis of Meynert
**len-fa**	lenticular fascicle	field H2 (441) ; area subthlamica tegmentalis, pars ventrolateralis; dorsal division of ansa lenticularis	lenticular fasciculus of telencephalon and nucleus of ventral field of subthalamus (61976; 77527)	fasciculus lenticularis and nucleus campi ventralis [H2] (A14.1.08.664; A14.1.09.521; A14.1.08.706)	fasciculus lenticularis	lenticular fasciculus; Forel H2 field; H2; area tegmentalis H2; pallido-thalamic fascicle; H2 field of Forel; dorsal division of ansa lenticularis; ventral field	faisceau lenticulaire; champ H2 de Forel; faisceau H2 du champ de Forel; faisceau lenticulaire de Forel; faisceau inférieur des faisceaux de Forel	area subthlamica tegmentalis, pars ventralis, pars ventrolateralis; fasciculus lenticularis hyopthalami; fasciculus pedunculi
**la-co**	lamina cornea	lamina cornea (n.a.)	n.a.				lame cornée (ependymal thickening)	lamina cinerea, infrachoroidea
**l-m-lam-len**	lateral medullary lamina of lenticular nucleus	lateral medullary lamina (234)	lateral medullary lamina of globus pallidus (62469)	lamina medullaris lateralis (A14.1.09.508)	lamina medullaris lateralis	external (outer) medullary lamina / between the putamen and pallidum	lame médullaire externe	lamina medullaris lateralis (externa, limitans); stria medullaris lateralis
**m-m-lam-len**	medial medullary lamina of lenticular nucleus	medial medullary lamina (235)	medial medullary lamina of globus pallidus (62470)	lamina medullaris medialis A14.1.09.510)	lamina medullaris medialis	mesial medullary lamina / between GPe and Gpi	lame médullaire interne	lamina medullaris medialis or interna; stria medullaris medialis
**olf-t**	olfactory tubercle	olfactory tubercle (262)	anterior perforate substance (61891)	tuberculum olfactorium (A14.1.09.433)	tuberculum olfactorium		tubercule olfactif; substance perforée antérieure	area olfactoria; tuberculum olfactorium
**pc**	posterior commissure	posterior commissure (484)	posterior commissure (62072)	commissura posterior (A14.1.08.416)	commissura posterior	PC	commissure blanche postérieure (CP)	commissura posterior or caudalis or dorsalis

**pgp**	pontes grisei pedunculares	peduncle of substantia nigra (n.a.)	n.a.			gray laminae between the fibers of the stratum intermedium pedunculi (pedunculus substantiae nigrae; kamm system; système en peigne); bridge substantia nigra and globus pallidus	n.a.; fibers= stratum intermedium de Meynert; couche dorsale du poied du pédoncule	kamm system
**p-plc-z**	posterior para lenticulocapsular zone	n.a.				posterior para lenticulocapsular area of reticular appearance	n.a.	
**p-subp-a**	posterior subpallidal area	n.a.				posterior subpallidal area of reticular appearance	n.a.	
**put**	putamen	putamen (230)	putamen (61834)	putamen (A14.1.09.507)	putamen		putamen	nucleus lenticularis, pars tertius
**retrolent-retic-z**	retrolenticular reticularoid zone	n.a.				retrolenticular reticularoid zone that limits laterally the Wernicke zone	segement retrolenticulaire de Déjerine	n.a.
**su-ot**	supraoptic tract	supraopticohypophysial tract (391)	dorsal supraoptic decussation (62051); ventral supraoptic decussation (62052)	commissura supraoptica dorsalis (A14.1.08.947); commissura supraoptica ventralis (A14.1.08.959)	commissurae supraopticae	includes the supraoptic commissures	comissures supraoptiques	commissurae supraopticae; dorsal and ventral
**str-ter**	stria terminalis	stria terminalis (286) & nuclei (dorsal nucleus,3225;…)	stria terminalis (61974)	stria terminalis (A14.1.09.275)	stria terminalis	mixed structure whith fibers and nuclei	strie terminale	stria terminalis or semicircularis or cornea
**th-fa**	thalamic fascicle	Forel's field H1; area subthlamica tegmentalis; pars dorsomedialis; part of ansa lenticulmaris	field h1 (62065) and nucleus of dorsal field of subthalamus (77526)	fasciculus thalamicus (A14.1.08.679) and nucleus campi dorsalis [H1] (A14.1.08.705)	fasciculus thalamicus	thalamic fasciculus; Forel H1 field; H1; area tegmentalis H1; H1 field of Forel; dorsal field	faisceau thalamique; champ H1 de Forel; faisceau H2 du champs de Forel; faisceau supérieur des faisecaux de Forel	area subthlamica tegmentalis dorsalis, pars dorsomedialis; fasciculus thalamicus hypothalami
**v-clau**	ventral claustrum	ventral endopiriform claustrum (3202)	n.a.	claustrum (A14.1.09.421)	claustrum ventrale	ventral part of the claustrum; endopiriform	avant-mur, claustrum	claustrum; claustrum temporale, substriatale and prae amygdalae

**zow**	zone of Wernicke	triangular area of Wernicke (2286)	n.a.			extended triangular area of Wernicke	zone ou champ de Wernicke	area triangularis; area or field of Wernicke; zona lateralis
**thalamus**								
**al-nu-th**	anterolateral nucleus of thalamus	n.a.				made of anteromedial (AM), anterodorsal (AD), and supranucleus lateropolaris (L.po) / ventral anterior (VA)	groupe nucléaire antérieur; noyau antérieur du thalamus	nucleus lateralis
**amd-nu-th**	anteromedial dorsal nucleus of thalamus	anterodorsal nucleus of the thalamus (303)	anterodorsal nucleus (62141)	nucleus anterodorsalis (A14.1.08.604)	nucleus anterodorsalis	nucleus anteroprincipalis (A.pr) or anteroventralis (AV)	groupe nucléaire antérieur; noyau antérieur du thalamus	nucleus medialis thalami; nucleus anteroventralis (AV); nucleus anteroprincipalis thalami
**ami-nu-th**	anteromedial intermediate nucleus of thalamus	anteromedial nucleus of the thalamus (304)	anteromedial nucleus (62142)	nucleus anteromedialis (A14.1.08.605)	nucleus anteromedialis	anteromedial nucleus (of thalamus)	groupe nucléaire antérieur; noyau antérieur du thalamus	nucleus anteroventralis; nucleus anteromedialis thalami
**amv-nu-th**	anteromedial ventral nucleus of thalamus	n.a.				nucleus fasciculosus (Fa); medioventral partially	groupe nucléaire antérieur; noyau antérieur du thalamus	nucleus medialis; nucleus fasciculosus thalami
**a-th-rad**	anterior thalamic radiation	anterior thalamic radiations (1726)	anterior radiation of thalamus (76976)	radiatio anterior thalami (A14.1.08.666)	radiatio anterior thalami; thalamo-frontal fibers	rostral peduncle of thalamus	contingent antérieur des radiations thalamiques ; stratum zonale; pédoncule antérieur de la couche optique	pedunculus rostralis, anterior, thalami; radiatio thalamo-frontalis
**cmp-nu-th**	centromedian, parvo, nucleus of intralaminar thalamus	centromedian nucleus (323)	centromedian nucleus (62165)	nucleus centromedianus (A14.1.08.618)	nucleus centromedianus	centromedian nucleus, pars parvocellular (posterior) (LaCM, pv)	centre médian de Luys; noyau central, médian, du thalamus	centri mediani
**cmm-nu-th**	centromedian, magno, nucleus of intralaminar thalamus	centromedian nucleus (323)	centromedian nucleus (62165)	nucleus centromedianus (A14.1.08.618)	nucleus centromedianus	centromedian nucleus, pars magnocellular (anterior) (LaCM, Mg)	centre médian de Luys; noyau central, médian, du thalamus	centri mediani

**dl-nu-th**	dorsolateral nucleus of thalamus	lateral dorsal nucleus (326)	lateral dorsal nucleus (62176)	nucleus dorsalis lateralis (A14.1.08.608)	nucleus dorsalis lateralis	nucleus dorso-oralis, intermedii, possibly dorso-caudalis; VA partially, ventral-lateral posterior (VLp) partially, possibly lateral posterior	noyau latéral postérieur	nucleus lateralis
**dm-nu-th**	dorsomedial nucleus of thalamus	not individualized				nucleus dorsal superficialis	noyau latéral dorsal ± antérieur	nucleus antero-dorsalis
**e-lam-th**	external lamina of thalamus	external medullary lamina (367)	n.a.	lamina medullaris lateralis (A14.1.08.660)	lamina medullaris lateralis	exterrnal (lateral) lamina of thalamus	lame/strie médullaire externe du thalamus	lamina medullaris lateralis, externus, thalami; stratum reticulatum
**hab**	habenula	habenula (294)	habenula (62032)	habenula (A14.1.08.003)	habenula	habenula (habenular nuclei)	habenula; ganglion de l'habenula	nucleus habenularis; ganglion habenulare
**il-nu-th**	intermediolateral nucleus of thalamus	nucleus zentrolateralis (n.a.)	n.a.			zentrolateralis (Z); VLa partially, VLp, VPLa partially	n.a.	nucleus lateralis; zentrolateralis
**i-th-ped**	inferior thalamic peduncle	inferior thalamic peduncle (371)	inferior radiation of thalamus (76980)	radiatio inferior thalami (A14.1.08.668)	radiatio inferior thalami	ventral thalamic peduncle, stalk / forms the ansa peduncularis combined with the ansa lenticulris	pédoncule thalamique inférieur (part of the ansa peduncularis); pédoncule inféro-interne du thalamus; pédoncule inférieur du thalamus	pedunculus ventralis or inferior or medialis or infero-internus thalami; radiatio thalamo-strialis
**lac-nu-th**	laminar caudal nucleus of thalamus	intralaminar nuclear group (317)	intralaminar nuclear group of thalamus (62021)	nuclei intralaminares thalami (A14.1.08.615)	nuclei intralaminares posteriores	posterior (CL); limitans (Li); paraventricular (PV)	noyaux intralaminiares; noyaux de la lame/strie médullaire interne	nuclei of the Lamina medullaris medialis
**lao-nu-th**	laminar oral nucleus of thalamus	intralaminar nuclear group (317)	intralaminar nuclear group of thalamus (central lateral, 62170)	nuclei intralaminares thalami (centralis lateralis, A14.1.08.16)	nuclei intralaminares anteriores	dorsomedial (central lateral, CL, nucleus; cucularis nucleus partially) and ventromedial (central medial, CeM; commissuralis partially)	noyau central médial du thalamus & noyau central latéral du thalamus	nuclei of the lamina medullaris medialis

**lg-b**	lateral geniculate body	lateral geniculate complex (352)	lateral geniculate body (62209)	corpus geniculatum laterale (A14.1.08.302)	corpus geniculatum laterale	dorsolateral geniculate body	corps genouillé latéral (métathalamus); corps genouillé externe	corpus geniculatum
**mg-b**	medial geniculate body	medial geniculate body (355)	medial geniculate body (62211)	corpus geniculatum mediale (A14.1.08.303)	corpus geniculatum mediale		corps genouillé médian (métathalamus)	corpus geniculatum
**m-nu-th**	medial nucleus of thalamus	medial dorsal nucleus (312)	medial dorsal nucleus (62156)	nucleus mediodorsalis (A14.1.08.622)	nucleus mediodorsalis	mediodorsal or dorsomedial nucleus of the thalamus; nucleus medialis dorsalis	noyau dorso-médian; noyau interne du thalamus	nucleus medialis
**mt-fa**	mammillothalamic fascicle	mammillothalamic tract (2438)	mammillothalamic tract (83849)	fasciculus mammillothalamicus (A14.1.08.671)	fasciculus mammillothalami cus	fascicle of Vicq d'Azyr; bundle of Vicq d'Azyr; mammillothalamic tract of the hypothalamus	faisceau mamillo-thalamique; faisceau de Vicq d'Azyr; faisceau thalamo-mamillaire	fasciculus mammillo-thalamicus, thalamo-mammillaris; fasciculus of Vicq d'Azyr
**pf-nu-th**	parafascicular nucleus of thalamus	parafascicular nucleus (324)	parafascicular nucleus (62166)	nucleus parafascicularis (A14.1.08.620)	nucleus parafascicularis	nucleus parafascicularis	noyau parafasciculaire du thalamus	centri mediani
**plg-b**	pre lateral geniculate body	pregeniculate nucleus (354)	ventral nucleus of lateral geniculate body (62215)	nucleus ventralis corporis geniculati lateralis (A14.1.08.806)	nucleus ventralis corporis geniculati lateralis	nucleus pregeniculatum	n.a.	nucleus praegeniculatus; nucleus accessorius; corpus geniculatum externum; griseum praegeniculatum
**pth-ret-z**	prethalamic reticularoid zone	n.a.				n.a.		
**pul**	pulvinar	pulvinar (328)	pulvinar (62178)	nuclei pulvinares (A14.1.08.610)	pulvinar	nucleus posterior; nucleus pulvenaris thalami	pulvinar	pulvinar thalami; nucleus posterior thalami
**sfl-th**	superficial lateral thalamus	reticular nucleus of the thalamus (365)	thalamic reticular nucleus (62026)	nucleus reticularis thalami (A14.1.08.638)	nucleus reticularis prethalami	reticular nucleus of thalamus (of Arnold); reticular nuclear group	zone grillagée d'Arnold & noyau réticulaire du thalamus	nucleus reticularis

**sfm-th**	superficial medial thalamus	reuniens nucleus (309)	reuniens nucleus (62153)	nucleus reuniens (A14.1.08.632)	nuclei periventriculares	midline and massa intermedia / that contains the nucleus endymalis or reuniens or medioventral, and parataenialis, paraventricularis, commissuralis rhomboidalis nuclei / midline nuclear complex of thalamus, or medial or subependymal or para-ependymal thalamus	groupe de la ligne médianne	nucleus mediani thalami
**str-med-th**	stria medullaris of thalamus	stria medullaris (298)	stria medullaris of thalamus (62080)	stria medullaris thalami (A14.1.08.106)	stria medullaris prethalami	stria medullaris thalami	strie médullaire; taenia thalami	stria habenularis; habenae; stria pinealis; taenia habenulae, thalami
**vcl-nu-th**	ventrocaudal lateral nucleus of thalamus	ventral posterolateral nucleus (344)	posterior ventrolateral nucleus of thalamus (84350)	nucleus ventralis posterolateralis (A14.1.08.641)	nucleus ventralis posterolateralis	ventral posterior lateral or posterolateral nucleus (VPL); ventral posterior medial (VPM), VPLp partially	noyau ventral postérieur latéral	nucleus ventralis postero-lateralis
**vcm-nu-th**	ventrocaudal medial nucleus of thalamus	ventral posteromedial nucleus (347)	ventral posteromedial nucleus (62202)	nucleus ventralis posteromedialis (A14.1.08.642)	nucleus ventralis posteromedialis	ventral posterior median nucleus (VPM); crescent; basal ventral medial, submedius; VPM, VPL partially	noyau ventral postérieur médian; noyau semi-lunaire de Flechsig; corpus cupuliforme	nucleus ventralis postero-medialis or arcuatus thalami
**vi-nu-th**	ventral intermediate nucleus of thalamus	oral part of ventral posterolateral nucleus (345)	oral part of ventral posterolateral nucleus (62205)	nucleus ventralis intermedius (A14.1.08.655)	nucleus ventralis posterolateralis	nucleus ventrointermedius (Vim); VLp (ventral part)	noyau ventral intermédiare (anterior part of the ventral posterior)	nucleus ventralis
**voa-nu-th**	ventrooral anterior nucleus of thalamus	ventral anterolateral nucleus of the thalamus (1816)	anteroventral nucleus (62143)	nucleus anteroventralis (A14.1.08.606)	nucleus ventralis anterior		noyau ventral latéral	nucleus ventralis
**vom-nu-th**	ventrooral medial nucleus of thalamus	ventral anterolateral nucleus of the thalamus (1816)	anteroventral nucleus (62143)	nucleus anteroventralis (A14.1.08.606)	nucleus ventralis anterior		noyau ventral latéral	nucleus ventralis
**vop-nu-th**	ventrooral posterior nucleus of thalamus	ventral anterolateral nucleus of the thalamus (1816)	anteroventral nucleus (62143)	nucleus anteroventralis (A14.1.08.606)	nucleus ventralis anterior		noyau ventral latéral	nucleus ventralis
